# Exposures associated with infection with *Cryptosporidium* in industrialised countries: a systematic review protocol

**DOI:** 10.1186/s13643-018-0731-8

**Published:** 2018-05-02

**Authors:** Caoimhe McKerr, Sarah J. O’Brien, Rachel M. Chalmers, Roberto Vivancos, Robert M. Christley

**Affiliations:** 1 NIHR Health Protection Research Unit in Gastrointestinal Infections, The University of Liverpool, Liverpool, UK; 2 NIHR Health Protection Research Unit in Emerging and Zoonotic Infections, The University of Liverpool, Liverpool, UK; 3 Cryptosporidium Reference Unit, Public Health Wales, Swansea, UK; 4 Field Epidemiology Services, Public Health England, Liverpool, UK; 5 Institute of Infection and Global Health, University of Liverpool, Waterhouse Building (2nd Floor, Block F), 1-5 Brownlow Street, Liverpool, L69 3GL UK

**Keywords:** Cryptosporidium, Protozoa, Outbreaks, Sporadic disease, Zoonoses, Gastrointestinal infection, Risk factors, Epidemiology, Parasite, Foodborne diseases, Waterborne diseases

## Abstract

**Background:**

*Cryptosporidium* is a protozoan parasite of humans and other animals worldwide and is one of the greatest contributors to human diarrhoeal illness. Transmission can occur indirectly via contaminated food or water, or directly via contact with animals or other infected people. Risk exposures are often identified from outbreak investigations, but a subset of cases remains unexplained, and sources for sporadic disease and pathways to infection are still unclear.

Given the few systematic syntheses of reported evidence in industrialised populations, the aim of this review is to consolidate the literature to describe exposures associated with human cryptosporidiosis in industrialised countries, specifically including the UK, and describe any differences between outbreak-associated and sporadic disease.

**Methods/design:**

Where relevant, methods will follow the recommendations made in the Cochrane Handbook for Systematic Reviews of Interventions. Three steps will be used to identify the literature including electronic database searching using PubMed, Scopus, Embase and Web of Science; reference list trawling; and an exploration of the grey literature. Screening of results will be undertaken by two reviewers using pre-defined criteria. Studies conducted in industrialised countries and reporting on human subjects will be included. All observational studies will be included where they report exposures and relevant quantitative results.

Data will be extracted using a standardised form. Study quality will be assessed using the ROBINS-I tool. Data will be summarised presenting the papers’ main findings including population under study, outcomes, and exposures, and whether these were considered outbreak or sporadic cases. A narrative summary will also be included. Where populations are appropriate, available data will be pooled in a meta-analysis combining the significant exposures across studies.

**Discussion:**

This review aims to consolidate the evidence for transmission routes and exposures for *Cryptosporidium* in industrialised countries, with particular reference to how these may apply to the UK. In addition, the review will seek to describe differences between outbreak and sporadic cases. This will help to identify those most vulnerable, highlighting pathways where interventions and public health response may be appropriate.

**Systematic review registration:**

PROSPERO number CRD42017056589.

**Electronic supplementary material:**

The online version of this article (10.1186/s13643-018-0731-8) contains supplementary material, which is available to authorized users.

## Background

*Cryptosporidium* is a protozoan parasite which can infect humans and other animals, and the most prevalent species identified in humans are *Cryptosporidium parvum* and *Cryptosporidium hominis* [1, 2]. *Cryptosporidium* is distributed worldwide and is suspected to be one of the greatest contributors to human diarrhoeal illness [3]. *Cryptosporidium* is reported in 1–3% of immunocompetent patients with diarrhoea in industrialised countries and 7–20% in developing countries [4–8]. The dissimilarities are probably driven by variation in exposure due to sanitation, infrastructure, and housing and health factors such as acquired immunity and nutrition. The highest prevalence is observed among children under 5 years old, in particular the under twos [3, 9]. The parasite has a complex life cycle and characteristics which favour the faecal-oral transmission route, which may facilitate outbreaks via person-to-person (*C. hominis* and *C. parvum*) or animal-to-person (*C. parvum*), as well as indirect transmission through ingestion of water and food contaminated with infectious oocysts [10].

Reported risk exposures for both *C. parvum* and *C. hominis* often overlap and include consumption of contaminated drinking water [11–15] and exposure to recreational waters [16–18] and food-related outbreaks (likely contaminated via water or by food handlers) [19–22].

*C. parvum* is frequently associated with exposure to farm animals [23, 24] due to its zoonotic nature and *C. hominis*, more anthropo-zoonotic, with person-to-person spread [25–28] and foreign travel [29]. Risk factors and associated exposures are often hypothesised/identified from outbreak investigations; however, outbreaks may only represent a small proportion of cases. Estimates in the United Kingdom (UK) suggest, of all cases reported to national surveillance in England & Wales, < 10% are likely to be linked to an identified outbreak [30]. However, the accuracy of the case numbers captured by surveillance may be imprecise [31, 32]. As a consequence, pathways may be under-reported and we cannot be certain that transmission routes for sporadic disease are the same as those which drive outbreaks [33]. Despite case-control studies which have investigated differences in risk for endemic and outbreak disease [34, 35], sources for sporadic disease and pathways to infection are still unclear and a substantial subset of reported cases remain unexplained.

### Previous reviews

A search of PubMed and the Cochrane Library revealed five previous systematic reviews which have synthesised evidence on risk factors for infection, all published between 2006 and 2016.

Two reports dealt with only immunocompromised populations: a review of C*ryptosporidium* prevalence in HIV/AIDS patients [36] and another assessing the treatment in immunocompromised patients [37]. A 2006 global review by Gualberto and Heller of drinking water sources found that unboiled water was associated with an increased risk of endemic cryptosporidiosis [38]. Another paper looked at seasonal patterns of five gastrointestinal pathogens together, including C*ryptosporidium*, in the Organisation for Economic Co-operation and Development (OECD) countries [39]. The paper hypothesised that environmental factors, e.g. land use, rainfall, temperature, and host characteristics, e.g. social contact, travel, and animal proximity, were drivers for seasonal patterns of cryptosporidiosis, and this was further buttressed by the existence of comparable evidence from New Zealand for other enteric pathogens [40]. However, these reviews were unable to report results by *Cryptosporidium* species, which may impact on risk factors, or investigate separately sporadic and outbreak-related cases for any variation in associations.

Given the absence of any systematic synthesis of reported evidence in the UK, and the few reviews in the rest of the industrialised countries, the aim of this review is to search the literature, including unpublished work, and describe the purported exposures associated with infection with *Cryptosporidium* in industrialised countries, specifically including the UK. In addition, there may be scope for a meta-analysis to support assessment of the available evidence and to explore differences that may exist in exposures or associations between sporadic and outbreak-related cases.

### Research question

In industrialised populations, what exposures are associated with human infection with *Cryptosporidium* and are these different for outbreak-associated and sporadic disease?

## Methods

To improve the transparency and completeness of the protocol, a copy of the Preferred Reporting Items for Systematic Reviews and Meta-Analyses for Protocols 2015 (PRISMA-P 2015) [41] checklist can be found in Additional file 1. This protocol is written following this checklist and guidance.

### Population

The review will include human populations only.

To avoid missing papers that may be useful to this review, a decision was made not to exclude key at-risk groups, where known, such as HIV/AIDS patients. The wealth of literature available indicates that these are well-studied groups and may act as good sentinels for the identification of transmission risks or pathways for immunocompetent populations. At the data collection and analysis stages, high-risk or highly susceptible populations, where known, can be separated for a more nuanced interpretation.

Searches will be restricted to reports from industrialised countries given that the literature suggests that transmission pathways and exposures, as well as susceptibility of populations, are different between these and countries with less infrastructure [42]. An industrialised country will be defined using OECD category of countries based on membership (Table 1) [43]. Where studies report results from a mix of industrialised and non-industrialised countries, and it is not possible to disentangle outcomes and exposures, the study will be excluded.

**Table 1:** Current membership—OECD

Australia	Japan
Austria	Korea
Belgium	Latvia
Canada	Luxembourg
Chile	Mexico
Czech Republic	Netherlands
Denmark	New Zealand
Estonia	Norway
Finland	Poland
France	Portugal
Germany	Slovak Republic
Greece	Slovenia
Hungary	Spain
Iceland	Sweden
Ireland	Switzerland
Israël	Turkey
Italy	United Kingdom
	United States

### Exposure

All exposures, including food, water, animal, environmental, and human, will be considered for inclusion.

### Outcome

Primary outcomes will include identifying exposures associated with *Cryptosporidium* infection and/or disease among both sporadic disease and outbreak-related cases. Outcomes among exposed populations will be compared to those in unexposed populations, where the study design allows. We are also interested in capturing molecular detail such as species where possible, as risk factors and exposures may vary.

### Inclusion/exclusion criteria (Table 2)

Only studies conducted in industrialised countries (as previously described) and reporting on human subjects will be included. All observational studies will be included where they report risk factors and relevant quantitative results. To allow us to capture the most relevant and robust information on risk factors at a population level, individual case reports will be excluded.

**Table 2:** Criteria for inclusion in the search

Inclusion criteria	Exclusion criteria
Any language—abstract (if available) in English	Cases known/defined as travel-related/acquired in non-industrialised country
Publication period—any	Individual case reports
Human cases	
All *Cryptosporidium* sp. including mixed	
Industrialised countries	
Known immunocompromised groups where risk factors are reported	
Known outbreaks	

Where the information is clearly communicated, we will exclude information describing cases who acquired their infection in a non-industrialised country and there is no further follow-up, for example, reporting on secondary spread. Where we cannot accurately determine country of infection, these will be excluded.

To capture any changes in incidence and factors associated with *Cryptosporidium* over time, there will be no limitation on publication period. We are also interested in capturing molecular detail, such as species, where possible, as risk factors and exposures for these may vary and this may be pertinent for comparisons of pathways and of value to the knowledge of zoonotic transmission routes.

There are no restrictions on language, provided the abstract can be made available in English for the first round of screening.

### Search strategy and terms

Where relevant, methods will follow the recommendations made in the “Cochrane Handbook for Systematic Reviews of Interventions” [44].

The search strategy proposed comprises three approaches, designed to collect as much relevant literature as possible from both peer-reviewed and grey sources.

The choice of databases was following advice from a University of Liverpool Medicine and Dentistry Liaison Librarian, as those deemed to be most relevant to the research question and likely to yield the highest number of relevant papers.

#### Step one—peer-reviewed literature

One reviewer (CMCK) will conduct electronic searches in the following databases of published literature considered most likely to yield the relevant papers:

- PubMed
- Web of Science
- Scopus
- Embase

The search terms were developed initially for PubMed and piloted in an iterative process ahead of commencing the review to ensure they successfully captured relevant papers. Where possible, terms were exploded to broaden the search. In the review, terms will be adapted as per the functionality of each database.

A more complete documented approach to developing the choices and finalising search terms is available on request.

Terms include the following categories:

- Organism terms: e.g. crypto*, C*ryptosporidium*, cryptosporidiosis
- Population term: e.g. “human”, patients, population,
- Transmission terms: e.g. transmission, risk factor, exposure, sporadic, infection, outbreak(s)
- Outcome terms: e.g. multivariate analysis, odds ratio, risk*, relative risk

Additional file 2 is an example of final search terms used for PubMed.

Search terms will be sought within the title, abstract, and keywords of the documents contained in each database. Filters within the three databases will be applied if required to restrict the results as appropriate according to inclusion criteria.

The publications captured using the final agreed search terms will be exported into reference managing software (Mendeley) and duplicates removed. The remaining publication titles will then be screened for relevance by two reviewers (CMCK and AW), using the inclusion and exclusion criteria.

#### Step two—hand-searching in papers

Reviewers (CMCK and AW) will search reference lists to identify any further literature or relevant publications not previously captured in the other strategies. The abstracts of any references considered potentially relevant will be sought and screened for inclusion using the inclusion and exclusion criteria.

#### Step three—accessing grey literature

One reviewer (CMCK) will access grey literature relevant to the review question using published online resources which may include bulletins and reports from relevant agencies, conference proceedings, and other relevant published outputs.

A search of Google Scholar (and any other relevant agencies’ sites, e.g. WHO) will be undertaken by entering the term ‘cryptosporidium’ with ‘risk factors’, ‘outbreak(s)’, ‘sporadic’, ‘endemic’, and/or ‘transmission’ into the application and reviewing the first 100 results for relevance. Using the same search terms and inclusion criteria, the same reviewer will carry out an additional search for unpublished theses work in the ProQuest database.

Abstracts (or relevant variations thereof) will be shared with the second reviewer (AW). Following agreement on inclusion, the work will be reviewed as per protocol.

To refine and clarify the inclusion criteria and search terms and ensure that the criteria can be applied consistently by all reviewers, the selection process will be piloted by applying criteria to a sample of papers.

### Abstract and paper selection

Following title selection, abstracts of the final included publications will be screened independently by two members of the review team (CMCK and AW) to ensure consistency in the application of the inclusion and exclusion criteria. Any discrepancies will be discussed and re-examined until an agreement is reached. A third reviewer is available for irreconcilable opinions on inclusion.

The full texts for all included works will be retrieved via the online library where possible and, if required, with the help of the University Liaison Librarian or by contacting authors. All full-text studies will be screened independently by the same reviewers (CMCK and AW) to ensure that they conform to the inclusion and exclusion criteria and discrepancies tackled as before.

Full-text papers which appear in a language other than English will be shared with colleagues in the Health Protection Research Unit (HPRU) and wider university teams for assistance with translation. An online translation tool (Google translate) will be used for initial screening where needed and where electronic papers are available for input.

Searching will cease when no further relevant and/or not previously identified work is being discovered.

### Data collection

A standardised data collection form will be developed in Covidence software. Each reviewer will be able to input data and update this as they each extract data from the papers. A minimum dataset of information from each paper will be extracted and recorded in duplicate, by each reviewer and, where information is available, will include variables outlined in Table 3.

**Table 3:** Minimum data set of information extracted from included papers

Bibliographic detail	Study detail
Name of reviewer	Study design
Date of extraction	Number of cases reported
Publication type	Age/sex cases/participants
Country of origin/language	Case definition (and any known co-infections)
Study title	Definition of exposure(s)
Names of authors	Definition of activities
Journal/source reference	Species identified
Year published	Incubation period
	Exposure window(s)
Study outcomes	General methodological
Number (%) exposed among groups	Confounders
Types of exposures	Likely biases
Comparator(s) (well controls, other infection)	
Selection and recruitment methods	
Availability of appropriate controls (from the same source population as the cases)	
Interview methods	
Effect measures (type and result)	

Studies will be allocated a unique identifier (automatically generated) and will be categorised according to the following groups:

- Included studies—studies that meet the eligibility criteria and are included in the review
- Excluded studies—studies that do not meet the eligibility criteria and are excluded from the review
- Studies awaiting classification—relevant studies that have been identified but cannot be assessed for inclusion until additional data or information are obtained
- Ongoing studies—studies that are ongoing and meet (or appear to meet thus far) the eligibility criteria

Disagreements will be discussed and, if required, rely on the input of a third reviewer as previously described.

### Assessing risk of bias

The ROBINS-I tool (Risk Of Bias In Non-randomized Studies - of Interventions) will be used as the framework for assessing quality of the studies. This instrument is well piloted and is specific to non-randomised study types [45]. The instrument provides an overall judgement on risk of bias using signalling questions across seven domains including bias, confounding, and missing data. Following assessment, each reviewer will label a study as ‘low’, ‘moderate’, ‘serious’, or ‘at critical’ risk of bias.

### Strategy for data synthesis

Search results and numbers of titles selected will be presented in the PRISMA 2009 flowchart [46].

In order to accurately report on the content of papers and to explore relationships between disease outcomes and risk factors, data will be summarised in a table presenting the main findings of each paper individually, including population under study, outcomes (infection with *Cryptosporidium sp*.), exposures, and general results (rates, prevalence, number of cases, odds, relative risks). A narrative summary of the characteristics and quality of the papers will also be included, alongside, and in the context of the strength of evidence results from ROBINS-I.

#### Meta-analysis

A certain level of heterogeneity is expected between studies which may include outcomes measured, population groups, type of study, and measures of association. Following these results, and a discussion about comparability of studies reported, a decision will be made regarding moving forward with a meta-analysis.

Where the populations are appropriate, and study quality allows, data will be pooled in a meta-analysis combining the significant exposures, and categories, across studies and presented as a summary of effects in their individual groupings, for example, ORs or RRs. Forest plots will be created for each exposure category (where paper numbers are high enough to retain validity) and examined to identify heterogeneity. Odds or risk of exposure among cases of *Cryptosporidium* will be presented according to the study design and outcome measured.

The summary measure and *I*^2^ statistic will be used to assess heterogeneity in the studies and will inform the use of meta-analysis techniques and the choice of a fixed or random effects model. Values of 30 to 60%, 50 to 90% and 75 to 100% will be used to denote moderate, substantial, and considerable levels of heterogeneity according to the Cochrane Handbook for Systematic Reviews of Interventions [44].

Data analyses will be carried out using RevMan, MS Access and Stata v12.0.

#### Data analysis plan

The data analysis will include a description of the cases and putative risk factors/exposures in each study, including the overall proportion of studies which report each exposure and the number of times a transmission pathway or risk factor is associated with illness.

Where possible, analyses of subgroup data may include:

- Outbreak vs non-outbreak disease
- Urban vs rural residence/populations
- Region of world
- *Cryptosporidium* species/genotype (e.g. *C. parvum* and *C. hominis*)
- Age groups of cases/non-cases
- Study design (such as cross-sectional, prevalence studies with risk factors, case-control, cohort, and other observational study designs, outbreak investigations, or surveillance analyses with risk factor information)

Aggregated study data by subgroup will be reported according to data type (e.g. mean and SD and percentages, ratios) and outcome measures (e.g. incidence, odds ratios, and relative risks). Studies will be further grouped by outcome measurement for consistency; studies reporting odds ratios will be aggregated separately to those reporting relative risk, for example. Exposures will be defined as per the paper under review, but where possible, they will be grouped into categories to allow for meaningful exposure group analyses. Categories are likely to include environmental exposures, water, animal exposures, exposure to a case, etc., and may also, where possible, include settings such as home, hospital, or nursery. Where data and number of papers allow these will be sub-grouped as much as possible.

Where data are missing or not reported in disaggregate form, the authors may be contacted in order to assist with further analyses. If the data allow, a more granular grouping of the studies may be undertaken to accurately address the research question.

#### Interpretation of findings

Given that we have not included any element of study design as part of the selection criteria for inclusion, interpretation of findings will begin with a description of the publication bias funnel plots where numbers of papers are sufficient. Discussions will include an exploration of all the strengths and weaknesses of the studies and a summary of the quality of evidence, using the Grading of Recommendations, Assessments, Development and Evaluation approach [47]. Most of the initial studies will likely be classed a priori as ‘low’ due to being observational in nature but may be upgraded after assessment of various domains of the tool, including bias, effect size, and precision. Papers will then be assigned a final grade for the quality of evidence as ‘high’, ‘moderate’, ‘low’, or ‘very low’ for all the critically important outcomes. Results will be reported using summary tables.

Interpretations of measures of effect may be stratified by study quality, and aggregated analyses of measures of effect will be assessed in the context of the populations under study.

### Dissemination

The protocol and the report will be prepared for peer-review publication.

The review will form part of a larger project submitted in partial fulfilment of a Doctor of Philosophy degree at the University of Liverpool.

Where appropriate, data may be presented as conference proceedings.

## Discussion

Many of the putative risk factors for cryptosporidiosis can have high exposure proportions and cases often report multiple risk factors, so well-designed studies are key in trying to elucidate clear pathways for transmission. More accurate understanding of the drivers behind continued apparent sporadic cryptosporidiosis has implications for public health intervention, control, and targeted treatment. This systematic review aims to describe the epidemiology and transmission of *Cryptosporidium* infection in industrialised countries, with particular reference to how this may apply to the UK. In addition, the review will seek to describe differences between outbreak and sporadic cases, investigating changes in prevalence and patterns among species and subtypes over time, and explore mechanisms for transmission of disease.

The results of this review will help support current knowledge and add to the evidence base on transmission pathways and risks for cryptosporidiosis, identifying those vulnerable and highlighting pathways where interventions may be of use.

The review will also help inform the development and direction of an analytical study as part of a PhD project.

## Additional files

Additional file 1: PRISMA-P (Preferred Reporting Items for Systematic review and Meta-Analysis Protocols) 2015 checklist: recommended items to address in a systematic review protocol. (PDF 170 kb)

Additional file 2: Search terms. (PDF 108 kb)

## Abbreviations

AIDS: Acquired immunodeficiency virus
GRADE: Grading of Recommendations, Assessments, Development and Evaluation
HIV: Human immunodeficiency virus
NIHR: National Institute for Health Research Health Protection Research Unit
OECD: Organisation for Economic Co-operation and Development
OR: Odds ratio
PHE: Public Health England
PHW: Public Health Wales
PRISMA-P: Preferred Reporting Items for Systematic Reviews and Meta-Analyses for Protocols
ROBINS-I: Risk Of Bias In Non-randomized Studies - of Interventions
RR: Relative risk/risk ratio
UK: United Kingdom
