# Supplementary material for: Exposures associated with infection with Cryptosporidium in industrialised countries: a systematic review protocol
Source: Syst Rev. 2018 May 2;7:70. doi: 10.1186/s13643-018-0731-8 (PMC5932784; doi:10.1186/s13643-018-0731-8)
Supplement: Supplementary file 2 — Search terms. (PDF 108 kb) [file 13643_2018_731_MOESM2_ESM.pdf]

Question components and search terms	Term type		Boolean operator
	Free	Mesh	
Population: cases of cryptosporidiosis	X		
1 cryptospor*			AND
2 humans		X	
3 1&2			
Exposure: risk factor(s)			
4 epidemiolog*	X		
5 risk factors	X		
6 exposure	X		OR
7 transmission	X		
8 association	X		
9 or/4-8			
Outcome: study endpoints			
10 cohort	X		
11 case-control	X		
12 “case control”	X		
13 case-crossover	X		OR
14 “disease outbreaks”		X	
15 meta-analysis	X		
16 longitudinal	X		
17 ecological	X		
18 or/10-17			
